# iTRAQ-Based Proteomic Analyses of Regulation of Isothiocyanate and Endogenous Selenium Metabolism in Broccoli Sprouts by Exogenous Sodium Selenite

**DOI:** 10.3390/foods12071397

**Published:** 2023-03-25

**Authors:** Xiaolan Quan, Yuwei Cheng, Zhengfei Yang, Jia Yang, Weiming Fang, Yongqi Yin

**Affiliations:** 1 College of Food Science and Engineering, Yangzhou University, Yangzhou 225127, China; 2 Yangzhou Center for Food and Drug Control, Yangzhou 225009, China

**Keywords:** broccoli sprouts, isothiocyanate, endogenous selenium, sodium selenite, proteomic

## Abstract

Broccoli sprouts have high isothiocyanate and selenium accumulation capacity. This study used a combination of methods, including physiological and biochemical, gene transcription and proteomic, to investigate the isothiocyanate and endogenous selenium accumulation mechanisms in broccoli sprouts under exogenous sodium selenite treatment during germination. Compared with the control, the sprouts length of broccoli sprouts under exogenous selenium treatment was significantly lower, and the contents of total phenol and malondialdehyde in 6-day-old broccoli sprouts were substantially higher. The contents of isothiocyanate and sulforaphane in 4-day-old were increased by up-regulating the relative expression of genes of *UGT74B1*, *OX-1*, and *ST5b*. The relative expression of *BoSultr1;1*, *BoSMT*, *BoHMT1*, and *BoCOQ5-2* genes regulating selenium metabolism was significantly up-regulated. In addition, 354 proteins in 4-day-old broccoli sprouts showed different relative abundance compared to the control under selenium treatment. These proteins were classified into 14 functional categories. It was discovered that metabolic pathways and biosynthetic pathways of secondary metabolites were significantly enriched. The above results showed that exogenous selenium was beneficial in inducing the accumulation of isothiocyanate and selenium during the growth of broccoli sprouts.

## 1. Introduction

Broccoli, an edible plant in *Brassica*, is rich in a variety of functional, active ingredients beneficial to human health, such as isothiocyanates (ITCs), sulforaphane (SFN), glucosinolates (GLs), phenolic compounds, etc. [1,2,3]. Shapiro et al. [4] showed that high consumption of broccoli could reduce the incidence of cancer, which is mainly related to ITCs [5]. In plants, ITCs are hydrolysates produced by hydrolysis of GLs under the action of myrosinase (MYR, EC3.2.1.147) [6], and ITCs are effective carcinogenic blockers, widely existing in broccoli [7]. They have many effects, such as antioxidation [8], anti-inflammatory [9], and prevention of cardiovascular disease [10]. In addition, SFN, an ITC has proven, has been found to have anticancer activity [11]. Broccoli sprouts are more suited to the enrichment of ITCs than mature broccoli because of the physiological and biochemical changes that occur during germination [12]. Moreover, abiotic stress is a widely used and effective strategy to promote the enrichment of ITCs in broccoli sprouts [13]. Based on the above factor, the enrichment of ITCs in broccoli sprouts under abiotic stress has aroused people’s interest.

Selenium (Se) is one of the trace elements found in the human body. It plays a crucial part in the regular function of the immune system and thyroid gland [14]. The primary source of Se in the human body is the consumption of plants high in Se. Therefore, a practical and effective way to produce foods containing Se is to enhance plants with Se at the proper concentration. Se supplementation can also promote the accumulation of various healthy secondary metabolites in plants [15,16]. Tian et al. [17] showed that the contents of SFN, anthocyanin, and flavonoids in broccoli sprouts increased markedly after Se treatment. In addition, broccoli sprouts can accumulate high Se, and the total Se content is dramatically increased under exogenous Se treatment [18]. Exogenous Se treatment increased the Se content in broccoli sprouts [17,18]. However, the molecular mechanism of exogenous Se in promoting the accumulation of ITCs and Se in broccoli sprouts is still unclear.

Based on the aforementioned issues, the proteome modifications caused by exogenous Se stress were investigated using the isobaric tag for the relative and absolute quantitative (iTRAQ) labeling approach. The findings of physiological and biochemical analyses, gene expression levels, and comparative proteomics analysis all contribute to a better understanding of the mechanisms affecting ITCs metabolism and Se metabolism in broccoli sprouts in response to Na_2_SeO_3_ treatment.

## 2. Materials and Methods

### 2.1. Plant Growth and Experimental Design

The broccoli seeds (*Brassica oleracea* L. var. Italica) were cleaned before being sterilized for 15 min with 1% (*v*/*v*) sodium hypochlorite. They were then soaked in distilled water at 30 °C for 4 h. The soaked seeds were then equally distributed over a clear case, covered with vermiculite, and allowed to germinate at 30 °C in an incubator with a 16 h light/8 h dark cycle. After a day of sprouting with distilled water, the treatments were carried out with different additives: (1) CK: distilled water; (2) Se: 0.10 mM Na_2_SeO_3_. Samples of 4-day-old and 6-day-old broccoli sprouts were taken randomly, then flash-frozen in liquid nitrogen and kept at −20 °C for later examination. The concentration of Na_2_SeO_3_ and the germination time depended on our pre-experiments.

### 2.2. Determination of Sprout Length, Fresh Weight, Malondialdehyde Content, and Total Phenolics Content

Thirty sprouts from each treatment were randomly selected to be measured in length with a micrometer and weighed in fresh weight (FW) with an electronic balance. The contents of malondialdehyde (MDA) and the total phenolics were determined following the protocol by Zhuang et al. [19] and Mencin et al. [20], respectively.

### 2.3. Determination of ITCs Content, Myrosinase Activity, Glucosinolates Content, and Sulforaphane Content

The content of ITCs was determined following the protocol by Ding et al. [5]. Absorbance was measured using a spectrophotometer (UV-7504C, Xinmao Instrument Co., Shanghai, China) at 365 nm. A standard curve was prepared with sulforaphane. The ITCs content was expressed as mg/100 g fresh weight of broccoli sprouts. MYR activity determination was conducted as described by Burow et al. [21]. Glucose content was determined by glucose kit (F006-1-1, Nanjing Jiancheng Biological Engineering Research Institute, Nanjing, China). Each minute was converted to 1 nmol glucose by MYR as one enzyme activity unit (U/mg protein). The GL content was measured according to Guo et al. [22]. Extraction and determination of SFN were performed according to Guo et al. [23]. The extracts were analyzed using a Thermo UHPLC U3000 Pump system (Thermo Fisher Scientific, San Jose, CA, USA) with a Gemini-NX C18 RP column (5 μm particle size, 3 × 250 mm, Phenomenex, Warsaw, Poland).

### 2.4. Determination of Inorganic Selenium and Organic Selenium Content

The separation of inorganic Se and organic Se was, according to Sun et al. [24]. The content of Se in the supernatant and precipitate measured based on the method of Lyi et al. [25] indicates the inorganic and organic Se content of the sample, respectively.

### 2.5. RNA Extraction and Quantitative Real-Time PCR Analysis

The total RNA isolated and reverse transcription was performed using the E.A.N.A.^TM^ Plant RNA Kit (R6827-01, OMEGA, Norcross, GA, USA) and the PrimeScript^TM^ RT Master Mix Kit (RR047A, Takara, Japan), respectively. Quantitative real-time PCR was performed on the cDNA samples using TB Green Premix DimerEraser^TM^ (RR091A, Takara, Japan). A list of the sequence-specific primers employed in this study is provided in Table 1.

### 2.6. Protein Extraction, Digestion, and iTRAQ Labeling

The total protein in 4-day-old broccoli sprouts was extracted, determined by using the Plant Total Protein Extraction Kit (PE0230, Sigma-Aldrich, St. Louis, MO, USA) and the Pierce^TM^ Coomassie Protein Assay Kit (23200, Thermo Fisher Scientific, Waltham, MA, USA), respectively. Following the procedure by Cheng et al. [26], the sample was reduced, alkylated, and then subjected to trypsin digestion. The iTRAQ 8-plex Kit (4381662, Sigma-Aldrich, St. Louis, MO, USA) was then used to label each sample individually by the manufacturer’s instructions. All samples were mixed and lyophilized finally.

### 2.7. LC-MS/MS and Data Analysis

The labeled samples were fractionated using a Thermo UHPLC U3000 Pump system (Thermo Fisher Scientific, San Jose, CA, USA) with a Gemini-NX C18 RP column (5 μm particle size, 3 × 250 mm, Phenomenex, Torrance, CA, USA). Detailed specific parameters for the liquid chromatography tandem mass spectrometry analysis are given in our previous research [26,27]. The raw tandem mass spectrometry files were processed using the Proteome Discoverer Software. Protein identification was performed using the uniport Arabidopsis thaliana database. The search parameters were as follows: trypsin was selected as the enzyme, with the tolerance set at one missed cleavage, a peptide allowance of 10 ppm, and an MS and MS/MS allowance of 0.02 Da. A protein had to contain at least two distinct peptides with a *p*-value less than 0.05 and a fold change larger than 1.5 or less than 0.67 to be classified as important differentially abundant proteins (DAPs) [28]. Identified proteins were annotated with their biological functions according to Kyoto Encyclopedia of Genes and Genomes (KEGG, http://www.kegg.jp/kegg/pathway.html, accessed on 10 July 2022) and the literature. Information on the DAPs was obtained from the universal protein resource (http://www.uniprot.org/, accessed on 10 July 2022). Pathway enrichment analysis was performed using DAVID6.8 (https://david.ncifcrf.gov/, accessed on 10 July 2022).

### 2.8. Statistical Analyses

The mean values ± standard deviation of the experimental data was expressed with three replications. One-way ANOVA and Tukey’s multiple tests were used to assess the data statistically, and a *p*-value of 0.05 was deemed significant. Relative gene expression was analyzed by the 2^−ΔΔCt^ method [29].

## 3. Results

### 3.1. Effect of Selenium on Growth Performance, Sprout Length, Fresh Weight, Malondialdehyde Content, and Total Phenolic Content of Broccoli Sprouts

**Figure 1:**
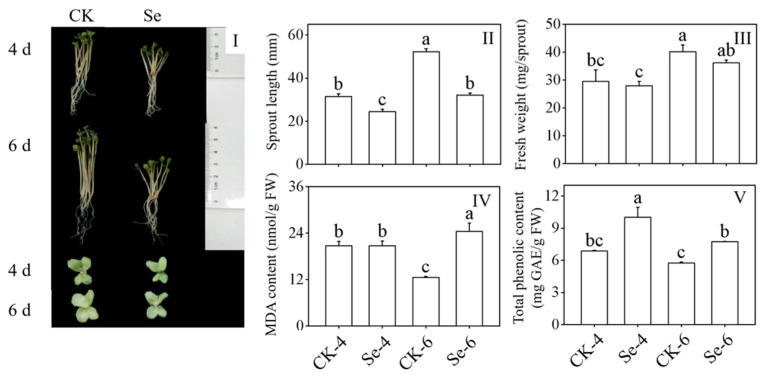
Effect of exogenous selenium on growth performance (**I**), sprout length (**II**), fresh weight (**III**), malondialdehyde content (**IV**), and total phenolic content (**V**) of broccoli sprouts during germination. Each data point represents the average of three independent biological replications (average ± SD). Lowercase letters reflected the significance of differences in indexes among treatments at different germination times using Tukey’s test (*p* < 0.05). For example, CK-4, Se-4, CK-6 and Se-6 indicated the 4- and 6-day-old broccoli sprouts treated with distilled water and 0.10 mM Na_2_SeO_3_, respectively.

The Na_2_SeO_3_ treatment considerably reduced the length of the broccoli sprouts and hindered their growth and development (Figure 1I,II) compared to the CK. However, it did not significantly affect their fresh weight (Figure 1III). MDA content, as a sign of membrane damage, significantly increased in 6-day-old broccoli sprouts treated with Na_2_SeO_3_ (*p* < 0.05) (Figure 1IV). To grow normally, total phenols with certain antioxidant capacities played a role, and their content increased dramatically by Na_2_SeO_3_ treatment (*p* < 0.05) (Figure 1V). The above facts showed that Na_2_SeO_3_ treatment hindered the growth and development of broccoli sprouts.

### 3.2. Effect of Selenium on ITCs Content, Myrosinase Activity, Glucosinolates Content, and Sulforaphane Content of Broccoli Sprouts

**Figure 2:**
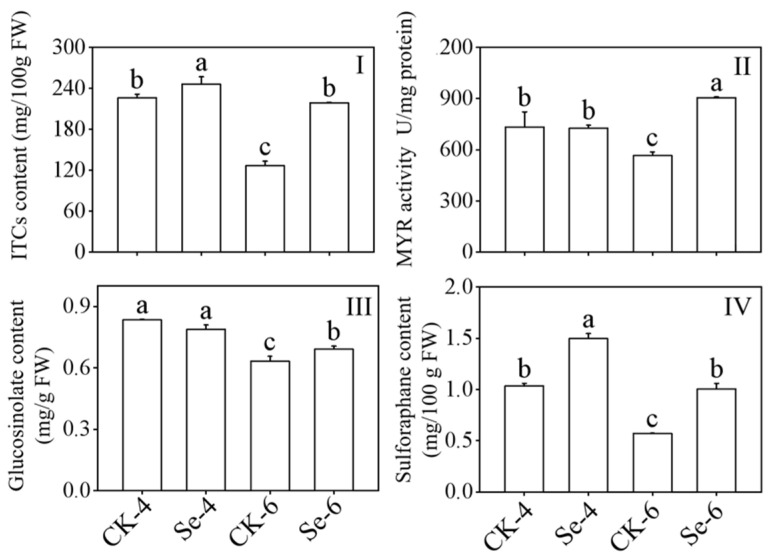
Effect of exogenous selenium on ITCs content (**I**), myrosinase activity (**II**), glucosinolate content (**III**), and sulforaphane content (**IV**) of broccoli sprouts during germination. Each data point represents the average of three independent biological replications (average ± SD). Lowercase letters reflected the significance of differences in indexes among treatments at different germination times using Tukey’s test (*p* < 0.05). For example, CK-4, Se-4, CK-6 and Se-6 indicated the 4- and 6-day-old broccoli sprouts treated with distilled water and 0.10 mM Na_2_SeO_3_, respectively.

GLs can be transformed by MYR into ITCs in plants. With Na_2_SeO_3_ treatment, the content of ITCs and SFN in broccoli sprouts all dramatically improved during germination as compared to the CK (*p* < 0.05), and the contents of ITCs and SFN in 6-day-old broccoli sprouts under Na_2_SeO_3_ treatment were 1.73 and 1.77 times more than the CK, respectively (Figure 2I,IV). The MYR activity and GLs content in 6-day-old broccoli sprouts increased significantly in 6-day-old broccoli sprouts (*p* < 0.05) (Figure 2II,III). In contrast to the CK, the GLs content dramatically dropped after 4 days of germination (*p* < 0.05) (Figure 2III). The above facts showed that Na_2_SeO_3_ treatment could promote the accumulation of ITCs in broccoli sprouts.

### 3.3. Effect of Selenium on Inorganic Selenium Content and Organic Selenium Content of Broccoli Sprouts

**Figure 3:**
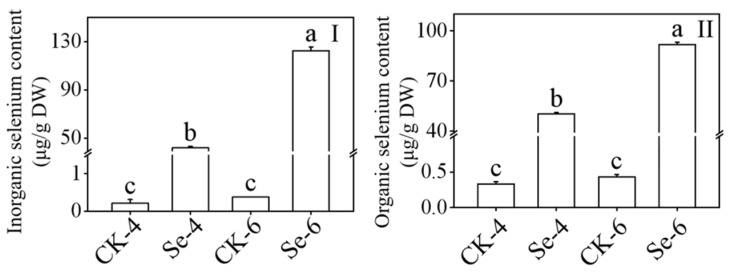
Effect of exogenous selenium on inorganic selenium content (**I**) and organic selenium content (**II**) of broccoli sprouts during germination. Each data point represents the average of three independent biological replications (average ± SD). Lowercase letters reflected the significance of differences in indexes among treatments at different germination times using Tukey’s test (*p* < 0.05). For example, CK-4, Se-4, CK-6 and Se-6 indicated the 4- and 6-day-old broccoli sprouts treated with distilled water and 0.10 mM Na_2_SeO_3_, respectively.

When broccoli sprouts were exposed to Na_2_SeO_3_ during germination, both the inorganic and organic Se contents drastically increased (*p* < 0.05) (Figure 3), and in 4-day-old broccoli sprouts treated with Na_2_SeO_3_, the contents of inorganic and organic Se were 199.19 and 153.06 times higher than the CK, respectively. According to the information, Na_2_SeO_3_ treatment can encourage Se accumulation in broccoli sprouts. 

### 3.4. Changes in Gene Expression of ITCs and Selenium Metabolism Key Enzyme in Broccoli Sprouts

**Figure 4:**
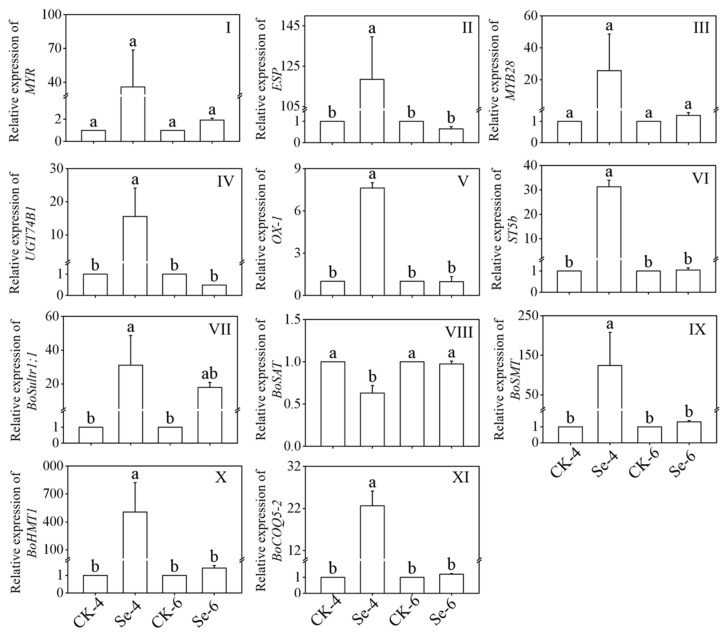
Changes of *MYR* (**I**), *ESP* (**II**), *MYB28* (**III**), *UGT74B1* (**IV**), *OX-1* (**V**), *ST5b* (**VI**), *BoSultr1;1* (**VII**), *BoSAT* (**VIII**), *BoSMT* (**IX**), *BoHMT1* (**X**), and *BoCOQ5-2* (**XI**) relative expression in broccoli sprouts under selenium treatment during germination. Each data point represents the average of three independent biological replications (average ± SD). Lowercase letters reflected the significance of differences in indexes among treatments at different times using Tukey’s test (*p* < 0.05). For example, CK-4, Se-4, CK-6 and Se-6 indicated the 4- and 6-day-old broccoli sprouts treated with distilled water and 0.10 mM Na_2_SeO_3_, respectively.

Figure 4I–VI showed the relative expression of ITCs metabolism key enzyme in broccoli sprouts treated with exogenous Se. As the figures show, the expression of *ESP*, *UGT74B1*, *OX-1,* and *ST5b* was significantly induced by Se treatment of 4-day-old broccoli sprouts, which were 118.48, 15.58, 7.62, and 31.34 times more than that of CK, respectively. While the expression of *MYR* and *MYB28* showed no significant change under Na_2_SeO_3_ treatment (*p* > 0.05).

Figure 4 VII–XI showed the relative expression of the Se metabolism key enzyme in broccoli sprouts under Na_2_SeO_3_ treatment. After Na_2_SeO_3_ treated 4-day-old broccoli sprouts, the expressions of *BoSultr1;1*, *BoSMT*, *BoHMT1* and *BoCOQ5-2* were 31.05-, 123.93-, 505.17-, and 22.63-fold of that in the CK, respectively, while Na_2_SeO_3_ treatment significantly decreased the expression level of *BoSAT* (*p* < 0.05). Compared with the CK, the expression of *BoSAT*, *BoSMT BoHMT1,* and *BoCOQ5-2* showed no significant change in 6-day-old broccoli sprouts (*p* > 0.05).

### 3.5. iTRAQ Analysis and Identification of Differentially Abundant Proteins

**Figure 5:**
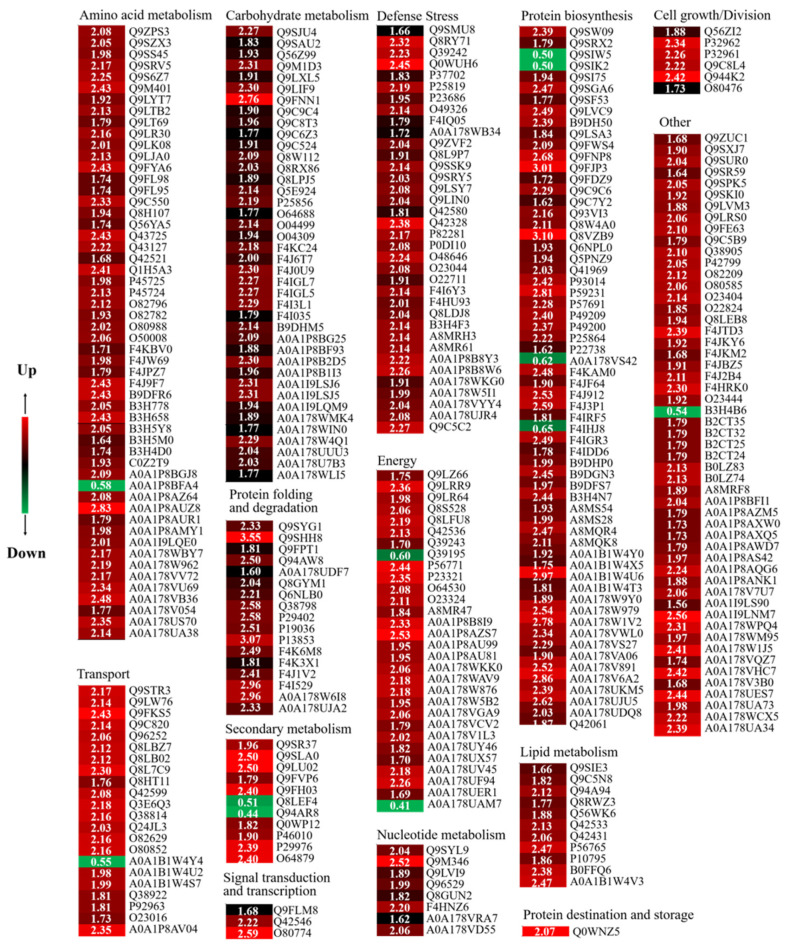
Heatmaps and cluster analysis of DAPs in Se/CK of 4-day-old broccoli sprouts. CK: control; Se: 0.10 mM Na_2_SeO_3_. Expression changes were made based on the log 2 conversion expression ratios of the proteins applying Gene Cluster 3.0 software. Visualize results using JAVA Treeview software. The numbers in the color scale are the changes in the abundance of the DAPs.

When the expression ratio is more than 1.50 or less than 0.67 and *p* < 0.05, it is considered DAPs [28]. The Se/CK samples in the present study had 354 DAPs, including 343 up-regulated and 11 down-regulated proteins (Table S1 and Figure 5).

**Figure 6:**
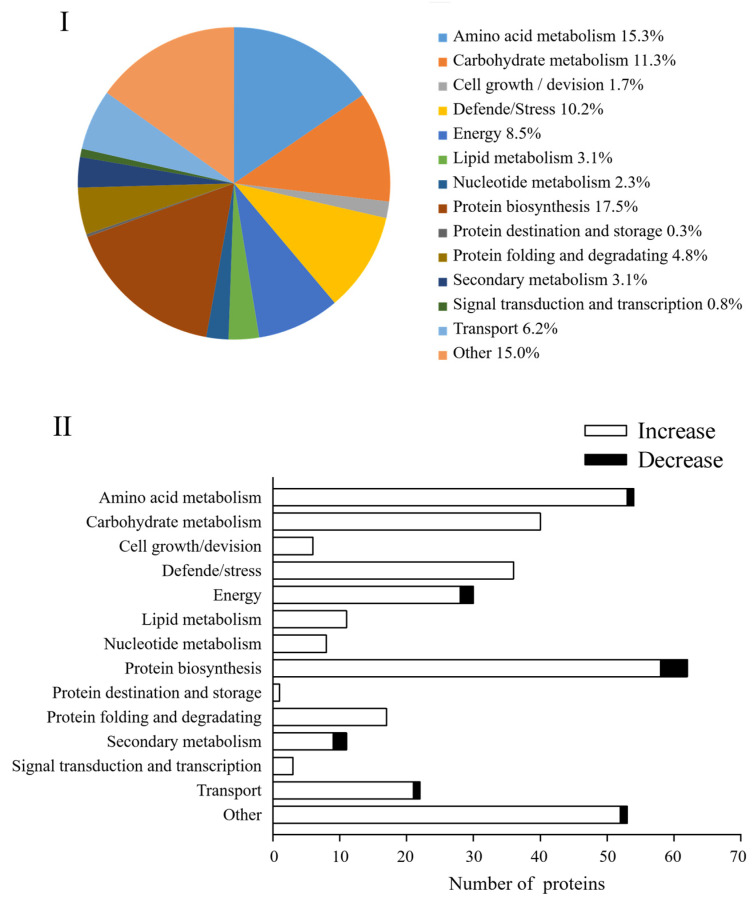
Comparison of proteomes of 4 d broccoli sprouts: The functions (**I**) and functional classification (**II**) of DAPs in Se/CK of 4-day-old broccoli sprouts. CK: control; Se: 0.10 mM Na_2_SeO_3_.

In this study, these DAPs could be classified into 14 functional classes based on the molecular functions listed on the UniProt and KEGG websites, i.e., amino acid metabolism, carbohydrate metabolism, cell growth/division, defende/stress, energy, lipid metabolism, nucleotide metabolism, protein biosynthesis, protein destination and storage, protein folding and degradation, secondary metabolism, signal transduction, and transcription, transport and other (Figure 6I). After four days of germination in Na_2_SeO_3_, the abundance of all DAPs in the carbohydrate metabolism, cell growth/division, defende/stress, lipid metabolism, nucleotide metabolism, protein destination and storage, protein folding and degradation, and signal transduction classes increased significantly (Figure 6II).

**Figure 7:**
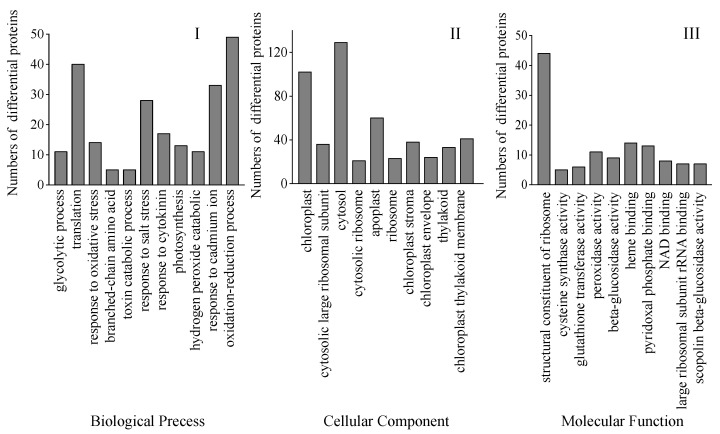
GO classification of all DAPs found in 4-day-old broccoli sprouts under exogenous selenium. Numbers of proteins in pathways of the biological process classification (**I**), cellular components (**II**), and molecular function classification (**III**).

Bioinformatics methods were used to evaluate these DAPs to get pertinent route data. To gather pertinent pathway data, these DAPs were analyzed utilizing bioinformatics methods. Biological process (BP), cellular component (CC), and molecular function (MF) were the three main gene ontology (GO) categories into which all the discovered peptides and DAPs under the Na_2_SeO_3_ treatment were categorized. The most frequent CC is chloroplasts and the cytosol, while the most frequent MF involves ribosome structural components. The most frequent BP was oxidation-reduction reactions and translation (Figure 7).

**Figure 8:**
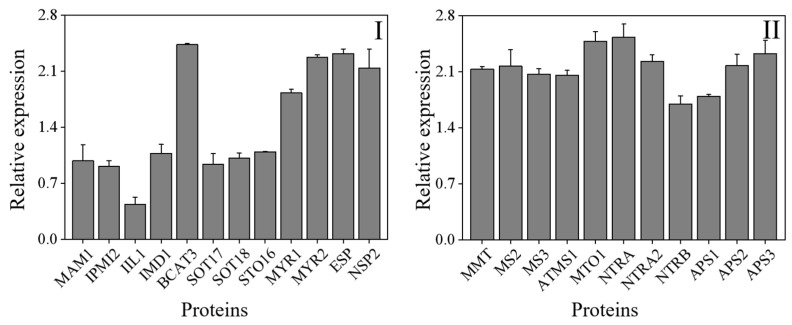
The differentia-regulated proteins involved in ITCs (**I**) and selenium (**II**) metabolism in 4-day-old broccoli sprouts on Se/CK. CK: control; Se: 0.10 mM Na_2_SeO_3_.

The KEGG database was used to do additional studies on the DAPs and examine the affected metabolic pathways. With a threshold of a *p*-value of less than 0.05, the pathway enrichment analysis discovered 22 KEGG pathways (Table 2). Most of the KEGG pathways were metabolic pathways, such as the synthesis of secondary metabolites, biosynthesis of antibiotics, and carbon metabolism. In the present study, a total of 23 differentia-regulated proteins involved in ITCs and selenium metabolism in 4-day-old broccoli sprouts were identified (Figure 8).

## 4. Discussion

The level of secondary metabolites in plants can be affected by abiotic stress [13]. For example, the production of sulfur-containing compounds GLs and SFN increased under sulfate stress [30]. While under the stress of exogenous Se, the growth and development of broccoli sprouts were significantly inhibited, and the length of the sprouts was significantly shortened (Figure 1I,II), which may be due to the production of many cellular structure-destroying components [31]. The MDA content, a sign of membrane damage, increased significantly in 6-day-old broccoli sprouts in this study (Figure 1IV). Total phenols with specified antioxidant capabilities were required for optimal growth, and their concentration increased considerably after Na_2_SeO_3_ treatment (Figure 1V). When plants are stressed, the GLs are hydrolyzed by MYR [32], and the ITCs content increased significantly under Se stress compared with the control in this study (Figure 2I). Gui et al. [33] also showed that Se could participate in vital physiological and metabolic processes and improve the antioxidant defence system in plants. Moreover, 343 DAPs out of 354 DAPs were up-regulated, and all DAPs associated with stress were up-regulated to ensure broccoli sprout development (Figure 5). In addition, the concentration of selenium also affects the growth of broccoli sprouts. A low concentration of Se can promote the growth of plants, while a high concentration of Se will inhibit the growth of sprouts [31]. Therefore, it may be that the concentration of Se used in this experiment was too high, which inhibited the growth and development of broccoli sprouts. 

S and Se are members of the VI–A elemental family of chalcogens. Thus, they have similar physical and chemical characteristics and share a common metabolic pathway in plants. Selenite treatment is beneficial to the biosynthesis of GLs and promotes the accumulation of Se in plants [34]. In our study, exogenous Se treatment enhanced the content of ITCs and SFN in broccoli sprouts (Figure 2I,IV). While the GLs content in 4-day-old broccoli sprouts decreased significantly (Figure 2III), it differed from the conclusion of Wang et al. [34]. It could be because broccoli and cabbage are different species or the consumption of cysteine in broccoli sprouts treated with Na_2_SeO_3_, as McKenzie et al. [35] showed that cysteine is the S donor of GLs and Se will bind to cysteine in the process of metabolism in plants to form selenocysteine. The level of inorganic Se and organic Se in broccoli sprouts increased considerably after treatment with exogenous Se (Figure 3), consistent with Avila et al. [36]. 

The transcription levels of key genes related to ITCs and Se metabolism in broccoli sprouts under Na_2_SeO_3_ treatment were analyzed using qRT-PCR (Figure 4). *MYB28*, *OX*-1, and *ST5b* regulate the synthesis of GLs, *MYR* is involved in the hydrolysis of GLs to ITCs, while *ESP* is involved in the conversion of GLs to nitrile products, and *UGT74B1* regulates the production of SFN, which is an ITC [3,37]. In this study, Na_2_SeO_3_ treatment caused several alterations in the expression of genes associated with ITC production. Na_2_SeO_3_ up-regulated the gene expression of *OX-1* and *ST5b* while related to GLs metabolism in 4-day-old broccoli sprouts. Nevertheless, GLs content in broccoli sprouts has no significant change compared to the control, which may be related to the excessive concentration of selenite that consumed the S donor cysteine of GLs [35]. The up-regulation of *UGT74B1* promoted the accumulation of sulforaphane in 4-day-old broccoli sprouts. *BoSultr1;1* and *BoCOQ5-2* are involved in promoting the absorption of Se in plants, and *BoSMT, BoSAT*, and *BoHMT1* play an important role in transforming the inorganic Se to organic Se [38,39]. In our study, under the treatment of exogenous Se, *BoSultr1;1, BoSMT*, *BoHMT1* and *BoCOQ5-2* genes in broccoli sprouts were significantly up-regulated, which promoted the absorption and transformation of selenite by 4-day-old broccoli sprouts.

The proteomic study further clarified the mechanism of accumulation of ITCs and endogenous Se in 4-day-old broccoli sprouts treated with Na_2_SeO_3_. A total of 354 DAPs were found in the current study’s Se/CK comparison group, of which protein biosynthesis, amino acid metabolism, carbon metabolism, and defence-related DAPs accounted for 17.5%, 15.3%, 11.3%, and 10.2%, respectively. According to the GO functional analysis, the proteins related to chloroplast and cytosol were highly expressed, which was beneficial to the enrichment of endogenous Se in broccoli sprouts, for chloroplasts played a key role in the absorption and transformation of Se in plants [39]. In addition, ATP-sulfurylase plays a catalytic selenate role in the cytosol [40]. The biological pathway of expressed proteins has been described using KEGG pathway analysis. A total of 22 metabolic pathways were identified, most of which belonged to metabolic pathways, such as the synthesis of secondary metabolites, biosynthesis of antibiotics and carbon metabolism, etc.

As shown in Figure 8, methylthioalkylmalate synthase 1 (MAM1), isopropylmalate isomerase 2 (IPMI2), 3-isopropylmalate dehydratase large subunit (IIL1), 3-isopropylmalate dehydrogenase (IMD1), branched-chain-amino-acid aminotransferase 3 (BCAT3), cytosolic sulfotransferase 16 (STO16), cytosolic sulfotransferase 17 (SOT17), cytosolic sulfotransferase 18 (SOT18), myrosinase 1 (MYR1), myrosinase 2 (MYR2), epithiospecifier protein (ESP) and nitrilespecilier protein 2 (NSP2) play an important role in the formation of ICTs (Figure 8). The enzymes IPMI2 (A0A178VZE1), IIL1 (Q94AR8), IMD1 (Q5XF32), SOT16 (Q9C9D0), SOT17 (Q9FZ80) and CYP83B1 (O65782) were involved in the metabolism of aliphatic glucosinolates, while MAM1 (Q9FG67), BCAT3 (Q9M401) and SOT18 (Q9C9C9) were involved in the metabolism of indole GLs [13,41]. Among these enzymes, only the relative expression of BCAT3 was significantly up-regulated, while the enzymes MYR1 and MYR2 that hydrolyzed GLs were significantly up-regulated, which could also explain the drop of GLs content in 4-day-old broccoli sprouts treated with exogenous Se. According to the National Library of Medicine and the study of others [42,43], 11 enzymes involved in Se metabolism were identified (Figure 8). They were methionine S-methyltransferase (MMT, Q9LTB2), 5-methyltetrahydropteroyltriglutamate-homocysteine methyltransferase 2 (MS2, Q9SRV5), 5-methyltetrahydropteroyltriglutamate-homocysteine methyltransferase 3 (MS3, Q0WNZ5), 5-methyltetrahydropteroyltriglutamate-homocysteine methyltransferase 1 (ATMS1, O50008), pyridoxal phosphate (PLP)-dependent transferases superfamily protein (MTO1, A0A178VB36), thioredoxin reductase (NTRA, A0A1P8AZS7), hioredoxin reductase 2 (NTRA2, Q39242), tnitrilase 1 (NTRB, Q39243), ATP-sulfurylase 1 (APS1, B2CT25), ATP-sulfurylase 2 (APS2, A0A178WAV9), ATP-sulfurylase 3 (APS3, A0A1P8B8I9). After exogenous Se treatment, the relative expression of these enzymes was dramatically up-regulated compared to the control, which was consistent with the result of the significant increase in organic Se and inorganic Se content.

## 5. Conclusions

Exogenous Se decreased the sprouts length of broccoli sprouts, increased the content of MDA, and hindered the growth and development of broccoli sprouts. However, from another perspective, the total phenolic content increased under exogenous Se treatment, and the abundance of ITCs and selenium metabolism-related proteins increased by up-regulating the expression level of ITCs and selenium metabolism genes and promoting the enrichment of ITCs and endogenous selenium, thereby increasing the value of broccoli sprouts.

## Figures and Tables

**Table 1:** The primer sequence of ITCs and Se metabolism genes in broccoli sprouts.

Primer	Forward Primer (5′-3′)	Reverse Primer (5′-3′)
*Actin*	CTGTTCCAATCTACGAGGGTTTCT	GCTCGGCTGTGGTGGTGAA
*MYB28*	AGACTGCGATGGACTAACTACCTAAA	CCGACCACTTGTTTCCACGA
*UGT74B1*	CAAAGACGATAAAGGCTACGGC	TCCCAAAGGAACCAAACGAA
*ST5b*	CCGACACTACCTTACCGAACCA	CGTGAGGAAAAGAGGCGATG
*OX-1*	GTGGACATTATACCGAACCTTACG	TGTGGACTTCTTTGGCGACCT
*MYR*	AAGGTCATCAGGGAGAAGGGTG	TGTTTGGCAGGGTTCTTAGTGG
*ESP-F*	ACATTTGGGACCAGGGACG	TTTCCATACACGGTGGCAGTC
*BoSultr1;1*	GATTCTGCTGCAAGTGACGA	ACGCGAATGATCAAGATTCC
*BoSAT1;1*	ATATCCATCCAGCAGCGAAG	CTGTCTCCGCAAGCTTTACC
*BoHMT1*	TTCAGGAATGCCTTGAAACC	TTAGCTTTTCCGTCCCACAC
*BoSMT*	GATCAACTGTACCCCTCCAAG	TCCCAACTCCTGTGTTTTCC
*BoCOQ5-2*	AAGGAAAGACTCGTTGGGAAG	TCCTAAACGCAACATCACCC

**Table 2:** Pathway enrichment analysis of differential proteins in 4-day-old broccoli sprouts. C: control; Se: 0.10 mM Na_2_SeO_3_.

Pathway ID	Pathway	Input Number	*p*-Value
		Se/C	Se/C
ath00195	Photosynthesis	12	0.0007
ath00250	Alanine, aspartate and glutamate metabolism	8	0.0061
ath00260	Glycine, serine and threonine metabolism	10	0.0056
ath00270	Cysteine and methionine metabolism	12	0.0057
ath00290	Valine, leucine and isoleucine biosynthesis	5	0.0198
ath00380	Tryptophan metabolism	8	0.0048
ath00450	Selenocompound metabolism	10	2.82 × 10^−8^
ath00460	Cyanoamino acid metabolism	10	0.0016
ath00480	Glutathione metabolism	10	0.0273
ath00620	Pyruvate metabolism	9	0.0395
ath00630	Glyoxylate and dicarboxylate metabolism	14	2.69 × 10^−5^
ath00710	Carbon fixation in photosynthetic organisms	11	0.0011
ath00920	Sulfur metabolism	15	7.49 × 10^−10^
ath00940	Phenylpropanoid biosynthesis	19	0.0003
ath01100	Metabolic pathways	132	9.41 × 10^−11^
ath01110	Biosynthesis of secondary metabolites	81	1.48 × 10^−7^
ath01130	Biosynthesis of antibiotics	52	2.01 × 10^−10^
ath01200	Carbon metabolism	45	1.23 × 10^−14^
ath01210	2-Oxocarboxylic acid metabolism	9	0.0202
ath01230	Biosynthesis of amino acids	37	6.95 × 10^−10^
ath03010	Ribosome	44	4.03 × 10^−9^

## Data Availability

The data presented in this study are available on request from the corresponding author.

## Supplementary Materials

The following supporting information can be downloaded at: https://www.mdpi.com/article/10.3390/foods12071397/s1, Table S1: Differentially abundant proteins in 4-day-old broccoli sprouts under exogenous selenium treatments.
