# Supplementary material for: iTRAQ-Based Proteomic Analyses of Regulation of Isothiocyanate and Endogenous Selenium Metabolism in Broccoli Sprouts by Exogenous Sodium Selenite
Source: Foods. 2023 Mar 25;12(7):1397. doi: 10.3390/foods12071397 (PMC10093868; doi:10.3390/foods12071397)
Supplement: Supplementary file 1 [file foods-12-01397-s001.zip › foods-2290366-supplementary.pdf]

Table S1 Differentially abundant proteins in 4-day-old broccoli sprouts under exogenous selenium treatments

Accession No.[a]	Description[b]	Se/C	Cove[c]	MW (kDa)[d]	TpId[e]
**Carbohydrate metabolism**					
Q9SJU4	Fructose-bisphosphate aldolase 1, chloroplastic	2.27±0.13	21.55	42.9	6.58
Q9SAU2	D-ribulose-5-phosphate-3-epimerase	1.83±0.24	5.69	29.99	8.07
Q56Z99	Alpha-xylosidase (Fragment)	1.93±0.45	10.96	8.08	5.38
Q9M1D3	Citrate synthase 5, mitochondrial	2.31±0.20	7.76	51.69	6.67
Q9LXL5	Sucrose synthase 4	1.91±0.07	2.85	92.94	6.57
Q9LIF9	Beta-glucosidase 19	2.30±0.11	2.47	59.98	6.65
Q9FNN1	Pyruvate kinase	2.76±0.18	2.16	54.94	6.29
Q9C9C4	Enolase 1, chloroplastic	1.90±0.06	3.77	51.44	6.13
Q9C8T3	Ribulose-phosphate 3-epimerase	1.96±0.10	7.93	24.1	6.1
Q9C6Z3	Pyruvate dehydrogenase E1 component subunit beta-2, chloroplastic	1.77±0.21	2.46	44.22	6.35
Q9C524	Probable fructokinase-6, chloroplastic	1.91±0.28	7.03	41.45	5.77
Q8W112	Beta-D-glucan exohydrolase-like protein	2.09±0.20	15.71	67.88	9.13
Q8RX86	Alpha-galactosidase 2	2.03±0.12	4.8	44.01	7.64
Q8LPJ5	Isocitrate dehydrogenase [NADP], chloroplastic/mitochondrial	1.89±0.14	5.98	54.16	7.97
Q5E924	Glyceraldehyde-3-phosphate dehydrogenase GAPCP2, chloroplastic	2.14±0.01	7.38	44.82	8.62
P25856	Glyceraldehyde-3-phosphate dehydrogenase GAPA1, chloroplastic	2.19±0.1	26.52	42.46	7.75
O64688	Pyruvate dehydrogenase E1 component subunit beta-3, chloroplastic	1.77±0.21	2.46	43.99	5.8
O04499	2,3-bisphosphoglycerate-independent phosphoglycerate mutase 1	2.14±0.07	5.92	60.54	5.53
O04309	Jacalin-related lectin 35	1.94±0.06	6.21	48.47	5.26
F4KC24	Xylose isomerase	2.18±0.19	27.18	32.39	7.75
F4J6T7	Putative alpha-xylosidase 2	2.00±0.01	2.3	97.39	6.62
F4J0U9	Dicarboxylate diiron protein, putative (Crd1)	2.30±0.27	3.01	39.15	6.44
F4IGL7	Fructose-bisphosphate aldolase	2.27±0.13	18.65	33.3	6.8
F4IGL5	Fructose-bisphosphate aldolase	2.27±0.13	20.05	41.78	6.28
F4I3L1	Phosphoglycerate kinase	2.29±0.03	14.81	42.59	5.54
F4I035	RNA 3'-terminal phosphate cyclase/enolpyruvate transferase, alpha/beta	1.79±0.23	3.07	52.37	5.99
B9DHM5	Pyruvate, phosphate dikinase	2.14±0.16	9.14	95.33	5.52
A0A1P8BG25	Glycosyl hydrolase family protein	2.09±0.20	13.96	77.11	9.32
A0A1P8BF93	Beta-D-xylosidase 4	1.88±0.04	6.87	67.23	7.12
A0A1P8B2D5	Beta glucosidase 15	2.30±0.09	2.25	40.18	7.94
A0A1P8B1I3	Malic enzyme	1.96±0.17	5.39	52.89	6.58

A0A1I9LSJ6	Citrate synthase	2.31±0.20	7.68	52.37	6.67
A0A1I9LSJ5	Citrate synthase	2.31±0.20	5.69	46.81	6.29
A0A1I9LQM9	Mannose-binding lectin superfamily protein	1.94±0.06	4.71	31.96	5.55
A0A178WMK4	VI2	1.89±0.37	2.17	45.52	5.08
A0A178WIN0	PDH-E1 BETA	1.77±0.21	2.46	44.23	6.35
A0A178W4Q1	Phosphoglycerate kinase	2.29±0.03	12.55	49.91	8.27
A0A178UUU3	Glucose-6-phosphate isomerase	2.04±0.12	3.26	67.01	5.69
A0A178U7B3	Alpha-galactosidase	2.03±0.12	4.46	47.23	7.64
A0A178WLI5	PDH-E1 BETA	1.77±0.21	3.26	33.75	5.9
**Amino acid metabolism**					
Q9ZPS3	Glutamate decarboxylase 4	2.08±0.34	7.1	55.97	6.34
Q9SZX3	Argininosuccinate synthase, chloroplastic	2.05±0.04	4.66	53.81	6.67
Q9SS45	Phenylalanine ammonia-lyase 4	1.98±0.17	1.98	76.87	6.27
Q9SRV5	5-methyltetrahydropteroyltriglutamate--homocysteine methyltransferase 2	2.17±0.20	18.43	84.53	6.51
Q9S6Z7	Bifunctional L-3-cyanoalanine synthase/cysteine synthase D1	2.25±0.08	3.4	34.27	5.34
Q9M401	Branched-chain-amino-acid aminotransferase 3, chloroplastic	2.43±0.02	2.42	44.94	8.1
Q9LYT7	3-isopropylmalate dehydratase small subunit 2	1.92±0.11	5.14	27.19	6.87
Q9LTB2	Methionine S-methyltransferase	2.13±0.44	4.3	118.64	5.63
Q9LT69	D-3-phosphoglycerate dehydrogenase 3, chloroplastic	1.79±0.01	1.7	62.08	8.24
Q9LR30	Glutamate--glyoxylate aminotransferase 1	2.16±0.04	16.84	53.27	6.89
Q9LK08	3-hydroxyisobutyryl-CoA hydrolase-like protein 4, mitochondrial	2.01±0.20	2.87	45.71	6.46
Q9LJA0	Putative inactive cysteine synthase 2	2.13±0.01	15.96	19.63	7.93
Q9FYA6	Branched-chain-amino-acid aminotransferase 5, chloroplastic	2.43±0.02	2.41	45.55	7.87
Q9FL98	Glutathione S-transferase family protein	1.74±0.10	2.57	40.59	5.59
Q9FL95	At5g45020/K21C13_21	1.74±0.10	2.77	37.32	6.33
Q9C550	2-isopropylmalate synthase 2, chloroplastic	2.33±0.19	4.6	68.09	6.37
Q8H107	Dihydrolipoyllysine-residue succinyltransferase component of 2-oxoglutarate dehydrogenase complex 2, mitochondrial	1.94±0.16	1.51	50.03	9.09
Q56YA5	Serine--glyoxylate aminotransferase	1.74±0.07	8.48	44.18	7.83
Q43725	Cysteine synthase, mitochondrial	2.43±0.17	9.3	45.79	8.18
Q43127	Glutamine synthetase, chloroplastic/mitochondrial	2.22±0.08	13.26	47.38	6.87
Q42521	Glutamate decarboxylase 1	1.68±0.01	6.97	57.03	5.53
Q1H5A3	Glutamate dehydrogenase	2.41±0.10	9.73	44.5	6.86
P45725	Phenylalanine ammonia-lyase 3	1.98±0.12	2.02	76.19	6.51
P45724	Phenylalanine ammonia-lyase 2	2.13±0.05	1.95	77.81	6.46

O82796	Phosphoserine phosphatase, chloroplastic	2.12±0.25	3.39	32.3	6.38
O82782	1-(5-phosphoribosyl)-5-[(5-phosphoribosylamino)methylideneamino] imidazole-4-carboxamide isomerase, chloroplastic	1.93±0.10	2.63	33.34	7.06
O80988	Glycine dehydrogenase (decarboxylating) 2, mitochondrial	2.02±0.02	0.77	113.7	6.65
O50008	5-methyltetrahydropteroyltriglutamate-homocysteine methyltransferase 1	2.06±0.06	23.27	84.3	6.51
F4KBV0	Transcriptional coactivator/pterin dehydratase	1.71±0.06	5.7	20.9	7.39
F4JW69	Phenylalanine ammonia-lyase	1.98±0.17	2.01	76.63	6.51
F4JPZ7	Peptidase M20/M25/M40 family protein	1.79±0.14	3.82	48.71	5.35
F4J9F7	Cysteine synthase	2.43±0.17	9.26	46.06	9.06
B9DFR6	Cysteine synthase	2.43±0.17	9.24	46.09	8.37
B3H778	Arginosuccinate synthase family	2.05±0.04	5.11	49	7.72
B3H658	Branched-chain-amino-acid aminotransferase	2.43±0.02	2.43	44.7	8.1
B3H5Y8	Glycine cleavage system P protein	2.05±0.09	1.74	106.16	6.99
B3H5M0	Monodehydroascorbate reductase	1.64±0.07	4.16	47.56	5.34
B3H4D0	Glutathione S-transferase family protein	1.74±0.10	3.37	31.11	5.24
C0Z2T9	1-(5-phosphoribosyl)-5-[(5-phosphoribosylamino)methylideneamino] imidazole-4-carboxamide isomerase, chloroplastic	1.93±0.50	3.59	24.7	8.73
A0A1P8BGJ8	Glutamate-1-semialdehyde-2,1-aminomutase	2.09±0.07	24.09	43.74	5.72
A0A1P8BFA4	Glutathione S-transferase family protein	0.58±0.03	3.35	31.14	5.4
A0A1P8AZ64	Glutamate decarboxylase	2.08±0.34	6.42	62	7.53
A0A1P8AUZ8	2-isopropylmalate synthase 1	2.83±0.22	5.26	59.93	7.05
A0A1P8AUR1	Class I glutamine amidotransferase-like superfamily protein	1.79±0.08	3.19	40.34	7.05
A0A1P8AMY1	3-phosphoserine phosphatase	1.98±0.36	3.58	31	6.96
A0A1I9LQE0	ATP-dependent caseinolytic (Clp) protease/crotonase family protein	2.01±0.20	3.49	37.79	8.05
A0A178WBY7	Glycine cleavage system H protein	2.17±0.10	5.42	17.89	5.19
A0A178W962	D-3-phosphoglycerate dehydrogenase	2.19±0.32	4.33	66.41	6.13
A0A178VV72	Glycine cleavage system H protein	2.17±0.10	5.45	17.94	5.34
A0A178VU69	Cysteine synthase	2.34±0.11	9.95	41.63	8.02
A0A178VB36	MTO1	2.48±0.12	2.13	59.88	6.87
A0A178V054	Cysteine synthase	1.77±0.07	26.09	33.78	6.14
A0A178US70	Phospho-2-dehydro-3-deoxyheptonate aldolase	2.35±0.09	4.14	56.11	8.78
A0A178UA38	Acyl-coenzyme A oxidase	2.14±0.13	1.01	77.43	8.29
**Lipid metabolism**					
Q9SIE3	At2g22230/T26C19.11	1.66±0.06	5.45	24.23	8.48
Q9C5N8	GDSL esterase/lipase At1g54020	1.82±0.15	2.15	41.77	7.94

Q94A94	Diaminopimelate decarboxylase 2, chloroplastic	2.12±0.10	3.89	54.13	6.67
Q8RWZ3	Probable acyl-CoA dehydrogenase IBR3	1.77±0.18	3.76	91.66	8.32
Q56WK6	Patellin-1	1.88±0.20	1.75	64.01	4.83
Q42533	Biotin carboxyl carrier protein of acetyl-CoA carboxylase 1, chloroplastic	2.13±0.01	9.29	29.59	9.06
Q42431	Oleosin 20.3 kDa	2.06±0.41	13.09	20.3	7.58
P56765	Acetyl-coenzyme A carboxylase carboxyl transferase subunit beta, chloroplastic	2.47±0.04	5.12	55.6	6.24
P10795	Ribulose bisphosphate carboxylase small chain 1A, chloroplastic	1.99±0.07	25.56	20.2	7.71
B0FFQ6	AccD (Fragment)	2.38±0.11	11.97	12.47	8.62
A0A1B1W4V3	Acetyl-coenzyme A carboxylase carboxyl transferase subunit beta, chloroplastic	2.47±0.04	5.12	55.57	6.24
**Nucleotide metabolism**					
Q9SYL9	50S ribosomal protein L13, chloroplastic	2.04±0.16	7.88	26.77	9.92
Q9M346	Fes1B	2.52±0.04	1.93	40.83	5.21
Q9LVI9	Dihydropyrimidine dehydrogenase (NADP(+)), chloroplastic	1.89±0.20	11.74	46.82	6.8
Q96529	Adenylosuccinate synthetase, chloroplastic	1.99±0.25	16.33	52.93	7.14
Q8GUN2	Adenylylsulfatase HINT1	1.82±0.24	19.73	15.99	7.2
F4HNZ6	Glyceraldehyde 3-phosphate dehydrogenase A subunit 2	2.20±0.14	28.71	34.31	6.64
A0A178VRA7	MORF6	1.62±0.01	3.45	26.35	8.9
A0A178VD55	GOX2	2.06±0.44	27.35	40.91	8.76
**Energy**					
Q9LZ66	Photosystem I reaction center subunit V, chloroplastic	1.75±0.01	10	17.08	9.57
Q9LRR9	ATP sulfurylase 4, chloroplastic	2.36±0.08	5.76	52.09	8.87
Q9LR64	Assimilatory sulfite reductase (ferredoxin), chloroplastic	1.98±0.01	7.63	71.91	8.31
Q8S528	(S)-2-hydroxy-acid oxidase GLO1	2.06±0.11	31.34	40.32	9.13
Q8LFU8	Photosystem II repair protein PSB27-H1, chloroplastic	2.19±0.17	17.82	18.82	9.88
Q42536	Aldehyde dehydrogenase family 2 member B7, mitochondrial	2.13±0.03	4.49	58.12	7.33
Q39243	Nitrilase 1	1.70±0.10	4.46	24.93	5.76
Q39195	Thioredoxin reductase 1, mitochondrial	0.60±0.01	12	39.6	7.36
P56771	Thioredoxin reductase 2	2.44±0.21	4.18	40.61	6.7
P23321	Oxygen-evolving enhancer protein 1-1, chloroplastic	2.35±0.14	38.55	35.12	5.66
O64530	Thiosulfate/3-mercaptopyruvate sulfurtransferase 1, mitochondrial	2.08±0.10	8.44	41.87	6.39
O23324	ATP-sulfurylase 3, chloroplastic	2.11±0.01	8.17	52	7.4

A8MR47	Sulfurtransferase	1.84±0.09	11.35	31.69	7.39
A0A1P8B8I9	Pseudouridine synthase/archaeosine transglycosylase-like family protein	2.33±0.17	7.45	57.49	8.51
A0A1P8AZS7	Thioredoxin reductase	2.53±0.16	4.43	38.23	8.12
A0A1P8AU99	Rhodanese homologue 2	1.95±0.07	3.01	29.43	5.8
A0A1P8AU81	Rhodanese homologue 2	1.95±0.07	2.76	32.13	6.8
A0A178WKK0	Sulfurtransferase	2.06±0.09	9.94	35.61	5.25
A0A178WAV9	ASA1	2.18±0.14	3.57	53.6	6.61
A0A178W876	PSAD-2	2.18±0.05	16.18	22.29	9.77
A0A178W5B2	Sulfurtransferase	1.95±0.07	2.52	34.7	5.27
A0A178VGA9	SOX	2.06±0.07	9.67	43.3	8.68
A0A178VCV2	APS1	1.79±0.02	12.31	51.43	6.81
A0A178V1L3	PDE332	2.02±0.04	10.68	25.65	9.06
A0A178UY46	PSBQA	1.82±0.20	10.31	23.78	9.64
A0A178UX57	PSII-Q	1.70±0.15	27.83	24.63	9.72
A0A178UV45	PSAD-1	2.18±0.05	15.87	22.58	9.77
A0A178UF94	SUPO1	2.26±0.13	10.81	43.48	6.46
A0A178UER1	PPa6	1.69±0.09	7.67	33.36	6.01
A0A178UAM7	ADK1	0.41±0.02	12.6	26.92	7.36
**Defense/Stress**					
Q9SMU8	Peroxidase 34	1.66±0.06	1.98	38.81	7.64
Q8RY71	Epithiospecifier protein	2.32±0.06	5.28	36.99	5.86
Q39242	Thioredoxin reductase 2	2.23±0.08	4.18	40.61	6.7
Q0WUH6	Catalase	2.45±0.14	27.44	56.73	7.42
P37702	Myrosinase 1	1.83±0.05	3.88	61.09	5.92
P25819	Catalase-2	2.19±0.10	34.15	56.9	7.12
P23686	S-adenosylmethionine synthase 1	1.95±0.06	24.17	43.13	5.82
O49326	Nitrile-specifier protein 2	2.14±0.24	4.46	51.18	5.76
F4IQ05	Peroxidase	1.79±0.19	5.41	33.25	7.68
A0A178WB34	Peroxidase	1.72±0.17	9.78	39.53	8.28
Q9ZVF2	MLP-like protein 329	2.04±0.03	11.92	17.59	5.55
Q8L9P7	Type 2 peroxiredoxin, putative	1.91±0.38	5.56	17.4	5.34
Q9SSK9	MLP-like protein 28	2.14±0.13	4.78	37.59	5.34
Q9SRY5	Glutathione S-transferase F7	2.03±0.01	3.83	23.58	6.62
Q9LSY7	Peroxidase 30	2.08±0.22	2.74	35.77	9.66
Q9LIN0	Major latex protein, putative	2.04±0.03	4.61	17.78	5.15
Q42580	Peroxidase 21	1.81±0.01	4.89	36.72	7.4
Q42328	Defensin-like protein 195	2.38±0.31	15.73	9.88	6.43
P82281	Thylakoid lumenal 29 kDa protein, chloroplastic	2.17±0.03	2.01	37.91	8.46
P0DI10	Peroxidase 1	2.08±0.22	2.77	35.6	9.25
O48646	Probable phospholipid hydroperoxide glutathione peroxidase 6, mitochondrial	2.24±0.18	8.62	25.57	9.35
O23044	Peroxidase 3	2.08±0.22	2.76	34.88	8.4

O22711	Peroxiredoxin-2D	1.91±0.04	5.56	17.46	5.54
F4I6Y3	MLP-like protein 28	2.14±0.13	6.43	27.53	5.77
F4HU93	Ascorbate peroxidase 1	2.01±0.01	31.73	27.5	6.29
Q8LDJ8	Major latex protein, putative	2.04±0.44	4.61	17.69	5.16
B3H4F3	MLP-like protein 28	2.14±0.13	4.62	19.33	5.29
A8MRH3	MLP-like protein 28	2.14±0.13	3.98	22.55	4.93
A8MR61	MLP-like protein 28	2.14±0.13	2.78	32.26	5.27
A0A1P8B8Y3	Ascorbate peroxidase 4	2.22±0.07	1.77	43.45	7.84
A0A1P8B8W6	Ascorbate peroxidase 4	2.26±0.10	2.46	31.02	6.83
A0A178WKG0	TPX2	1.91±0.04	5.56	17.4	5.54
A0A178W5I1	MEE6	1.99±0.02	35.2	27.54	6.13
A0A178VYY4	ZCE1	2.04±0.03	11.92	17.5	5.73
A0A178UJR4	Peroxidase	2.08±0.22	2.84	34.68	8.84
Q9C5C2	Myrosinase 2	2.27±0.38	4.02	62.69	7.44
**Protein biosynthesis**					
Q9SW09	40S ribosomal protein S10-1	2.39±0.72	8.47	19.44	9.67
Q9SRX2	60S ribosomal protein L19-1	1.79±0.06	15.42	24.59	11.36
Q9SIW5	40S ribosomal protein S25-1	0.50±0.14	9.17	12.12	10.58
Q9SIK2	40S ribosomal protein S25-2	0.50±0.14	9.26	12.06	10.7
Q9SI75	Elongation factor G, chloroplastic	1.94±0.04	10.86	86	5.6
Q9SGA6	40S ribosomal protein S19-1	2.47±0.05	53.85	15.82	10.08
Q9SF53	60S ribosomal protein L35-1	1.77±0.04	11.38	14.28	10.92
Q9LVC9	60S acidic ribosomal protein P3-2	2.49±0.04	15	11.86	4.51
B9DH50	AT5G52650 protein	2.39±0.72	8.38	19.47	9.51
Q9LSA3	60S ribosomal protein L30-3	1.84±0.10	29.46	12.27	9.69
Q9FWS4	50S ribosomal protein L31, chloroplastic	2.09±0.45	20.83	16.02	9.8
Q9FNP8	40S ribosomal protein S19-3	2.68±0.07	25.87	15.69	10.21
Q9FJP3	50S ribosomal protein L29, chloroplastic	3.01±0.03	12.72	19.37	10.51
Q9FDZ9	60S ribosomal protein L21-2	1.72±0.09	9.76	18.7	10.52
Q9C9C6	60S ribosomal protein L6-2	2.29±0.09	8.15	25.99	10.17
Q9C7Y2	Multiple organellar RNA editing factor 5, chloroplastic/mitochondrial	1.62±0.01	3.49	26.01	9.11
Q93VI3	60S ribosomal protein L17-1	2.16±0.04	20.45	19.89	10.11
Q8W4A0	Eukaryotic translation initiation factor 3 subunit M	2.11±0.25	5.04	46.76	5.12
Q8VZB9	60S ribosomal protein L10a-1	3.10±0.01	10.65	24.45	9.88
Q6NPL0	At4g09040	1.93±0.06	5.26	34.01	7.59
Q5PNZ9	At1g22780	1.94±0.13	17.76	17.53	10.54
Q41969	Eukaryotic translation initiation factor 2 subunit beta	2.03±0.38	7.46	30.64	7.2
P93014	30S ribosomal protein S5, chloroplastic	2.42±0.02	7.59	32.63	8.97
P59231	60S ribosomal protein L10a-3	2.81±0.23	10.6	24.52	9.82
P57691	60S acidic ribosomal protein P0-3	2.28±0.05	12.69	34.37	5.15
P49209	60S ribosomal protein L9-1	2.40±0.07	28.35	22	9.45

P49200	40S ribosomal protein S20-1	2.37±0.10	25	13.87	9.72
P25864	50S ribosomal protein L9, chloroplastic	2.22±0.03	13.2	22.12	9.67
P22738	60S ribosomal protein L3-2	1.62±0.10	9.49	44.52	10.11
A0A178VS42	MORF2	0.62±0.40	3.65	24.7	8.24
F4KAM0	60S acidic ribosomal protein family	2.48±0.03	20.22	8.94	5.29
F4JF64	Clp ATPase	1.90±0.18	26.71	102.18	6.21
F4J912	Ribosomal protein L5	2.53±0.01	8.95	21.53	5.86
F4J3P1	Ribosomal protein L14p/L23e family protein	2.59±0.02	48.8	13.4	10.05
F4IRF5	Translation elongation factor EF1A/initiation factor IF2gamma family protein	1.81±0.23	1.91	51.3	8.72
F4IHJ8	Ribosomal protein S25 family protein	0.65±0.01	9.35	11.93	10.68
F4IGR3	60S acidic ribosomal protein family	2.49±0.03	9.18	10.24	4.68
F4IDD6	tRNA synthetase beta subunit family protein	1.78±0.21	1.37	65.92	5.49
B9DHP0	AT4G27090 protein	1.99±0.27	22.39	15.5	10.04
B9DGN3	AT2G27710 protein	2.49±0.03	7.83	11.44	4.68
B9DFS7	AT4G00810 protein	1.97±0.11	15.93	11.3	4.32
B3H4N7	60S acidic ribosomal protein family	2.48±0.03	15.13	11.76	4.51
A8MS54	RNA-binding (RRM/RBD/RNP motifs) family protein	1.93±0.06	6.56	27.69	8.97
A8MS28	Ribosomal L27e protein family	1.99±0.07	11.45	15.03	10.15
A8MQR4	60S acidic ribosomal protein P0	2.47±0.05	11.15	30.6	4.84
A8MQK8	60S acidic ribosomal protein family	2.11±0.22	18.75	9.99	4.36
A0A1B1W4Y0	50S ribosomal protein L14, chloroplastic	1.92±0.12	21.31	13.57	9.29
A0A1B1W4X5	30S ribosomal protein S19, chloroplastic	1.75±0.05	11.96	10.6	10.77
A0A1B1W4U6	30S ribosomal protein S4, chloroplastic	2.96±0.01	7.46	23.23	10.33
A0A1B1W4T3	30S ribosomal protein S2, chloroplastic	1.81±0.17	3.81	26.89	9.66
A0A178W9Y0	40S ribosomal protein S12	1.89±0.01	19.44	15.37	5.55
A0A178W979	Emb2394	2.55±0.01	13.45	24.69	9.89
A0A178W1V2	Ribosomal protein	2.78±0.26	10.65	24.41	9.88
A0A178VWL0	RPL16A	2.34±0.10	17.58	20.82	9.92
A0A178VS27	Tubulin beta chain	2.29±0.10	34.3	50.71	4.83
A0A178VA06	HSP93-III	1.90±0.18	25.84	105.77	6.51
A0A178V891	RPL5A	2.47±0.05	11.3	34.34	9.31
A0A178V6A2	60S ribosomal protein L13	2.90±0.03	3.88	23.49	10.55
A0A178UKM5	Tubulin beta chain	2.39±0.02	34.3	50.57	4.83
A0A178UJU5	RPL5B	2.62±0.08	11.3	34.42	9.17
A0A178UDQ8	EMB1401	2.03±0.38	7.46	30.61	7.2
Q42061	Ribosomal protein PO (Fragment)	1.87±0.16	11.54	8.65	9.64
**Protein destination and storage**					
Q0WNZ5	5-methyltetrahydropteroyltriglutamate--homocysteine methyltransferase 3, chloroplastic	2.07±0.07	6.65	90.54	8.05
**Protein folding and degradation**					
Q9SYG1	17.4 kDa class III heat shock protein	2.33±0.14	7.1	17.35	8.29

Q9SHH8	Glutathione S-transferase U26	3.55±0.27	5.45	25.76	5.72
Q9FPT1	Ubiquitin carboxyl-terminal hydrolase 12	1.81±0.25	0.72	130.53	5.77
Q94AW8	Chaperone protein DNA 3	2.50±0.03	8.33	46.42	6.11
A0A178UDF7	Peptidylprolyl isomerase	1.60±0.06	18.28	61.57	5.29
Q8GYM1	Glutathione S-transferase U22	2.04±0.01	10.55	25.27	5.27
Q6NLB0	Glutathione S-transferase L1	2.21±0.04	3.8	27.15	5.1
Q38798	Calnexin homolog 2	2.58±0.09	8.65	60.45	4.83
P29402	Calnexin homolog 1	2.58±0.09	16.23	60.45	4.91
P19036	17.4 kDa class I heat shock protein	2.51±0.08	21.15	17.43	5.29
P13853	17.6 kDa class I heat shock protein 3	3.07±0.12	15.29	17.59	5.47
F4K6M8	Calreticulin family protein	2.49±0.02	8.52	61.38	4.86
F4K3X1	Ubiquitin-specific protease 12	1.81±0.25	0.81	115.08	5.68
F4J1V2	DNAJ homologue 3	2.41±0.05	10.2	37.65	8.18
F4I529	Calreticulin	2.96±0.09	9.2	49.11	5.8
A0A178W6I8	Calreticulin	2.96±0.09	9.18	48.5	4.6
A0A178UJA2	At5g22060	2.33±0.11	8.35	46.41	7.12
**Secondary metabolism**					
Q9SR37	Beta-glucosidase 23	1.96±0.14	5.73	59.68	6.92
Q9SLA0	Beta-glucosidase 14	2.50±0.07	1.64	54.98	8.48
Q9LU02	Beta-glucosidase 13	2.50±0.07	1.58	56.92	8.76
Q9FVP6	3-phosphoshikimate 1-carboxyvinyltransferase	1.79±0.23	2.88	55.8	6.79
Q9FH03	Beta-glucosidase 12	2.40±0.01	1.58	56.93	8.57
Q8LEF4	S-adenosylmethionine synthase 1	0.51±0.01	24.17	43.13	5.82
Q94AR8	3-isopropylmalate dehydrogenase	0.44±0.09	10.15	43.27	6.06
Q0WP12	Thiocyanate methyltransferase 1	1.82±0.06	11.79	27.39	4.64
P46010	Nitrilase 3	1.90±0.09	4.91	38	5.95
P29976	Phospho-2-dehydro-3-deoxyheptonate aldolase 1, chloroplastic	2.39±0.06	10.1	57.94	8.06
O64879	Beta-glucosidase 15	2.40±0.01	1.58	56.87	7.71
**Signal transduction and transcription**					
Q9FLM8	DNA-directed RNA polymerases II, IV and V subunit 12	1.68±0.01	13.73	5.89	8.18
Q42546	SAL1 phosphatase	2.22±0.10	12.46	37.54	5.17
O80774	AT2G34250 protein	2.59±0.14	2.11	52.06	9
**Transport**					
Q9STR3	Copia-like retroelement pol polyprotein	2.17±0.08	7.38	13.9	9.03
Q9LW76	Ras-related protein RABG3c	2.14±0.02	16.99	23	5.52
Q9FKS5	Cytochrome c1 2, heme protein, mitochondrial	2.43±0.32	9.77	33.67	6.18
Q9C820	Ras-related protein RABG3d	2.14±0.02	16.99	23.05	5.33
Q96252	ATP synthase subunit delta', mitochondrial	2.06±0.09	4.93	21.53	6.7
Q8LBZ7	Succinate dehydrogenase [ubiquinone] iron-sulfur subunit 1, mitochondrial	2.12±0.02	9.32	31.15	8.44
Q8LB02	Succinate dehydrogenase [ubiquinone] iron-sulfur subunit 2, mitochondrial	2.12±0.02	9.29	31.12	8.62

Q8L7C9	Glutathione S-transferase U20	2.30±0.02	8.76	24.99	5.78
Q8HT11	Photosystem II CP43 reaction center protein	1.76±0.21	3.5	43.97	7.59
Q42599	NADH dehydrogenase [ubiquinone] iron-sulfur protein 8-A, mitochondrial	2.08±0.26	9.46	25.49	5.41
Q3E6Q3	Ras-related small GTP-binding family protein	2.18±0.06	28.69	13.82	5.07
Q38814	Thiamine thiazole synthase, chloroplastic	2.16±0.03	13.47	36.64	6.23
Q24JL3	Thiosulfate/3-mercaptopyruvate sulfurtransferase 2	2.03±0.13	2.34	37.39	6.13
O82629	V-type proton ATPase subunit G2	2.16±0.16	16.04	11.74	5.58
O80852	Glutathione S-transferase F9	2.18±0.07	22.79	24.13	6.65
A0A1B1W4Y4	Photosystem I iron-sulfur center	0.55±0.06	82.72	9.03	7.08
A0A1B1W4U2	Photosystem I P700 chlorophyll a apoprotein A2	1.98±0.28	3.41	82.42	7.4
A0A1B1W4S7	Photosystem II protein D1	1.99±0.20	3.12	38.91	5.25
Q38922	Ras-related protein RABB1b	1.81±0.08	6.16	23.16	7.01
P92963	Ras-related protein RABB1c	1.81±0.08	6.16	23.15	7.42
O23016	Probable voltage-gated potassium channel subunit beta	1.73±0.15	10.98	36.52	7.42
A0A1P8AV04	2-isopropylmalate synthase 1	2.35±0.17	6	52.45	8.07
**Cell growth/Division**					
Q56ZI2	Patellin-2	1.88±0.20	1.46	75.96	4.92
P32962	Nitrilase 2	2.34±0.05	5.01	37.13	5.55
P32961	Nitrilase 1	2.26±0.11	2.89	38.13	6.28
Q9C8L4	Persulfide dioxygenase ETHE1 homolog, mitochondrial	2.22±0.06	12.93	32.31	7.01
Q944K2	Dolichyl-diphosphooligosaccharide--protein glycosyltransferase 48 kDa subunit	2.42±0.11	4.12	48.71	6.34
O80476	Methylesterase 2	1.73±0.10	5.7	29.65	5.34
**Other**					
Q9ZUC1	NADPH-dependent alkenal/one oxidoreductase, chloroplastic	1.68±0.03	14.25	40.96	8.35
Q9SXJ7	Chaperone protein ClpC2, chloroplastic	1.90±0.18	25.84	105.67	6.42
Q9SUR0	AT4G23670 protein	2.04±0.03	4.64	17.51	6.37
Q9SR59	Monodehydroascorbate reductase 3	1.64±0.09	4.08	48.33	5.27
Q9SPK5	Formate--tetrahydrofolate ligase	2.05±0.08	11.04	67.76	6.71
Q9SKI0	At2g10940/F15K19.1	1.92±0.02	7.56	29.63	9.39
Q9LVM3	YCF54	1.88±0.23	7.58	24.09	8.1
Q9LRS0	(S)-2-hydroxy-acid oxidase GLO2	2.06±0.44	32.97	40.28	8.97
Q9FE63	Profilin-5	2.10±0.06	9.92	14.03	4.81
Q9C5B9	Probable aldo-keto reductase 1	1.79±0.02	3.2	37.57	6.46
Q38905	Profilin-4	2.10±0.06	9.7	14.54	5.2

P42799	Glutamate-1-semialdehyde 2,1-aminomutase 1, chloroplastic	2.05±0.04	25.32	50.34	6.87
O82209	Copia-like retroelement pol polyprotein	2.12±0.04	7.38	13.84	9.04
O80585	Methylenetetrahydrofolate reductase 2	2.06±0.01	3.2	66.76	5.5
O23404	Pyruvate, phosphate dikinase 1, chloroplastic	2.14±0.16	8.31	105.07	6.38
O22824	Formin-like protein 2	1.85±0.05	0.78	98.26	7.9
Q8LEB8	Quinone oxidoreductase-like protein	1.94±0.17	16.83	32.71	6.16
F4JTD3	RNA binding Plectin/S10 domain-containing protein	2.39±0.72	10.07	16.36	9.48
F4JKY6	Alpha/beta-Hydrolases superfamily protein	1.92±0.01	3.2	43.03	8.19
F4JKM2	Cinnamyl alcohol dehydrogenase 5	1.68±0.07	2.8	38.69	5.44
F4JBZ5	Sulfite oxidase	1.91±0.03	12.75	32.66	7.03
F4J2B4	Proteasome component (PCI) domain protein	2.11±0.26	5.77	41.13	5.07
F4HRK0	Glyoxalase II 3	2.30±0.01	7.48	32.34	7.01
O23444	Putative epoxide hydrolase	1.92±0.41	2.24	61	8.51
B3H4B6	Ribosomal protein S25 family protein	0.54±0.11	21.5	11.92	10.68
B2CT35	APS1 (Fragment)	1.79±0.03	23.38	16.92	6.33
B2CT32	APS1 (Fragment)	1.79±0.03	23.38	16.89	6.33
B2CT25	APS1 (Fragment)	1.79±0.03	23.38	16.92	6.81
B2CT24	APS1 (Fragment)	1.79±0.02	23.38	16.9	6.33
B0LZ83	Phenylalanine ammonia-lyase	2.13±0.05	7.25	21.54	6.61
B0LZ74	Phenylalanine ammonia-lyase	2.13±0.05	7.18	21.74	6.61
A8MRF8	Sulfite oxidase	1.89±0.05	12.03	26.63	5.44
A0A1P8BFI1	UDP-glucuronic acid decarboxylase 3	2.04±0.11	47.28	35.41	7.17
A0A1P8AZM5	Alpha/beta-Hydrolases superfamily protein	1.71±0.05	3.11	24.32	5.6
A0A1P8AXW0	Acetone-cyanohydrin lyase	1.73±0.10	8.02	20.82	6.42
A0A1P8AXQ5	Acetone-cyanohydrin lyase	1.73±0.10	5.4	31.38	5.74
A0A1P8AWD7	NAD(P)-linked oxidoreductase superfamily protein	1.79±0.02	4.31	27.82	6.4
A0A1P8AS42	Glyoxylate reductase 2	1.97±0.03	19.73	31.26	5.54
A0A1P8AQG6	Aldehyde dehydrogenase 2B7	2.24±0.04	5.88	44.45	7.83
A0A178WCX5	NADH dehydrogenase [ubiquinone] iron-sulfur protein 8-B, mitochondrial	2.22±0.37	4.05	25.36	2.29
A0A178UA34	S10_plectin domain-containing protein	2.39±0.72	8.83	19.54	9.61
A0A1P8ANK1	PATELLIN 2	1.88±0.20	1.54	72.34	4.97
A0A178V7U7	Oleosin	2.06±0.41	17.61	14.88	7.42
A0A1I9LS90	Photosystem II subunit T	1.60±0.01	12.59	15.62	9.63
A0A1I9LNM7	Fes1B	2.56±0.07	2.16	36.48	5.31
A0A178WPQ4	COX6B	2.18±0.10	4.71	21.18	4.34
A0A178WM95	GR2	1.97±0.03	16.48	37.76	8.44
A0A178W1J5	MEE4	2.41±0.29	9.79	16.12	9.2
A0A178VQZ7	S-formylglutathione hydrolase	1.74±0.15	7.39	31.64	6.37
A0A178VHC7	GSA2	2.42±0.26	22.67	50.11	7.39
A0A178V3B0	CAD5	1.68±0.07	2.8	38.72	5.67

A0A178UES7	HSP18.2	2.44±0.08	9.94	18.12	7.42
A0A178UA73	OLEO2	1.98±0.39	10.05	21.27	9.36

Note: The result was expressed as mean of three biology replications. [a]Aceession No., Aceession number according to the UniProtKB database, accessed on 10 July 2022; [b]Description, Homologue protein name obtained using *Arabidopsis* database from the UniProtKB, accessed on 10 July 2022; [c]Cove, coverage; [d]MW, theoretical molecular weight; [e]TpI, theoretical isoelectric point; C: control; Se: 0.10 mM Na_2SeO_3
